# Normal fat mass cannot be reliably estimated in typical pharmacokinetic studies

**DOI:** 10.1007/s00228-020-03042-4

**Published:** 2020-11-18

**Authors:** Roeland E. Wasmann, Elin M. Svensson, Stein J. Schalkwijk, Roger J. Brüggemann, Rob ter Heine

**Affiliations:** 1 Department of Pharmacy, Radboud Institute for Health Sciences, Radboud University Medical Center, Geert Grooteplein-Zuid 10, 6500 HB Nijmegen, The Netherlands; 2 Department of Pharmaceutical Biosciences, Uppsala University, Uppsala, Sweden

**Keywords:** Normal fat mass, F*fat*, Fat-free mass, Population pharmacokinetics, Pharmacokinetic modeling, Non-linear mixed-effects modeling

## Abstract

**Purpose:**

An influential covariate for pharmacokinetics is (body) size. Recently, the method of estimation of normal fat mass (NFM) has been advocated. Here, the relative contribution of fat mass, estimated as a fraction fat (F*fat*), is used to explain differences in pharmacokinetic parameters. This concept is more and more applied. However, it remains unclear whether NFM can be reliably estimated in these typical studies.

**Methods:**

We performed an evaluation of the reliability of NFM estimation in a typical study size (*n* = 30), otherwise best-case scenario, by means of a pharmacokinetic simulation study. Several values of F*fat* were investigated.

**Results:**

In a typical pharmacokinetic study, high imprecision was observed for NFM parameter estimates over a range of scenarios. For example, in a scenario where the true value of F*fat* on clearance was 0.5, we found a 95% confidence interval of − 0.1 to 2.1, demonstrating a low precision. The implications for practice are that one could conclude that fat-free mass best describes the relationship of the pharmacokinetics with body size, while the true relationship was between fat-free mass and total body weight. Consequently, this could lead to incorrect extrapolation of pharmacokinetics to extreme body sizes.

**Conclusion:**

In typical pharmacokinetic studies, NFM should be used with caution because the *Ffat* estimates have low precision. The estimation of F*fat* should always be preceded by careful study design evaluation before planning a study, to ensure that the design and sample size is sufficient to apply this potentially useful methodology.

**Supplementary Information:**

The online version contains supplementary material available at 10.1007/s00228-020-03042-4.

## Introduction

Like humans, body size descriptors come in many shapes, with total body weight (TBW) and fat-free mass (FFM) currently being most accepted in the pharmacometric community [1]. Choosing correct size descriptors, which account for the presence of peripheral fat tissue, may be especially important when dosing a drug with a narrow therapeutic index (i.e., aminoglycosides), especially in populations with extreme body size. It has been argued that “traditional” descriptors are not adequate to account for the (relative) importance of peripheral fat tissue to describe pharmacokinetic changes with increasing body size. It assumes that the pharmacokinetic parameter of interest is related to either TBW or FFM.

Recently, the method of estimation of normal fat mass (NFM) has been advocated [2]. NFM is the sum of the calculated FFM (based on height, weight, and sex) and the estimated relative contribution of fat mass. This method is increasingly employed in relatively small studies [3–14]. NFM is defined by Eq. 1, where F*fat* is the estimate that reflects the relative contribution of fat mass. It can be used together with a standard NFM (NFM_STD_) and an allometric coefficient (*b*) to scale the population estimate (*θ*_pop_) to the individual parameter (*θ*) (Eq. 2) [15].

1 $$ \mathrm{NFM}=\mathrm{FFM}+\mathrm{F} fat\times \left(\mathrm{TBW}-\mathrm{FFM}\right) $$

2 $$ \theta ={\theta}_{\mathrm{pop}}\times {\left(\frac{\mathrm{NFM}}{{\mathrm{NFM}}_{STD}}\right)}^b $$

It follows that when F*fat* is 1, NFM equals TBW. When F*fat* is 0, NFM equals FFM and when F*fat* is (much) larger than 1, the value of NFM is determined by the amount of fat tissue relative to FFM. In fact, an F*fat* value of +∞ would represent a situation where fat tissue alone is driving changes in the parameter of interest. Finally, negative values of F*fat* are possible in a situation where, for example, clearance is impaired due to obesity. The use of the NFM method is currently reported in different ways: first, as an aid to investigate which size metric (i.e., TBW, FFM, or fat mass) is most suitable to describe the data after which one is chosen [3–10]; second, as an estimate where the value of F*fat* is reported [11–13]. And third, using a mixture of both where if an estimate is 0 or 1 then they are fixed to the respective value but when the estimates is between 0 and 1 then the actual value of the estimated is used and reported [14].

F*fat* estimates are considered drug-specific parameters that are also specific for the pharmacokinetic variable (i.e., clearance or volume of distribution) and can be used for pharmacokinetic extrapolation into populations with different body sizes or composition [2]. For this purpose, reliable estimates of F*fat* (i.e., reproducible point estimates with high precision) are needed. However, it remains unclear whether NFM, by estimation of F*fat*, can be reliably determined with a representative study size seen in the clinical setting. Therefore, we investigated the reliability of the estimates of several F*fat* values in typical pharmacokinetic studies, assuming an otherwise best-case scenario.

## Methods

### General approach

We performed two simulation studies. The purpose of the first study was to confirm that F*fat* can be reliably estimated when your study is large enough (*n* = 10,000). This study size should not be interpreted as a realistic size but as an approximation of an infinite number of subjects. The purpose of the second study was to investigate how well F*fat* can be estimated in a typically sized, but otherwise best-case scenario, pharmacokinetic study (*n* = 30). In this report, we will refer to these studies as “large” and “typical.”

### Virtual drugs

F*fat* can be defined as the contribution of fat mass to a drug clearance (CL) or volume of distribution (V) (Eq. 1). Here, we investigated 16 virtual drugs, consisting of all possible combinations of F*fat*: 0, 0.5, 1, and 5, for CL and V, respectively.

### Study populations

The best-case scenario for a reliable estimate of F*fat* requires a wide distribution in body size and specifically a wide distribution of the FFM and TBW difference (i.e., fat mass) [15]. We obtained realistic demographic data (weight, height, and sex) from the National Health and Nutrition Examination Survey (NHANES) database from data collected between 1999 and 2014 [16]. The FFM was predicted using weight, height, and sex according to the formula reported by Janmahasatian et al. [17]. For each virtual study, we randomly sampled per BMI group from the NHANES database to create three equally sized groups of the following: (1) normal weight (body mass index (BMI) 18.5–30 kg/m^2^); (2) obese (BMI 30–40 kg/m^2^); and (3) morbidly obese (BMI > 40 kg/m^2^), with 50% of the subjects being female.

### Pharmacokinetic model

All pharmacokinetic simulations and estimations were performed with NONMEM (v7.3) and Perl-Speaks-NONMEM (v4.7) [18, 19]. The virtual drug was administered as 1 mg intravenous bolus. Pharmacokinetic data were simulated using a one-compartment model with first-order elimination, CL of 0.693 L/h, and *V* of 1 L. Inter-individual variability (IIV) was 30% on both CL and *V*, and we added a proportional residual error of 15%. A 15% residual error is chosen to be in line with the most recent EMA and FDA guidelines for bioanalytical method validation, where a %CV in the precision of < 15% is defined as precise [20, 21]. The sampling schedule covered three half-lives: 0.25, 0.5, 0.75, 1, 1.25, 1.5, 2, and 3 h after dosing. None of the simulated pharmacokinetic data was below the limit of quantitation. NFM was standardized around a male with a TBW of 100 kg and an FFM of 60 kg according to Eq. 2. Fixed allometric coefficients of 0.75 and 1 for CL and *V*, respectively, were applied. The NONMEM model code is provided in the supplemental material.

### Stochastic simulation and estimation

**Fig. 1:**
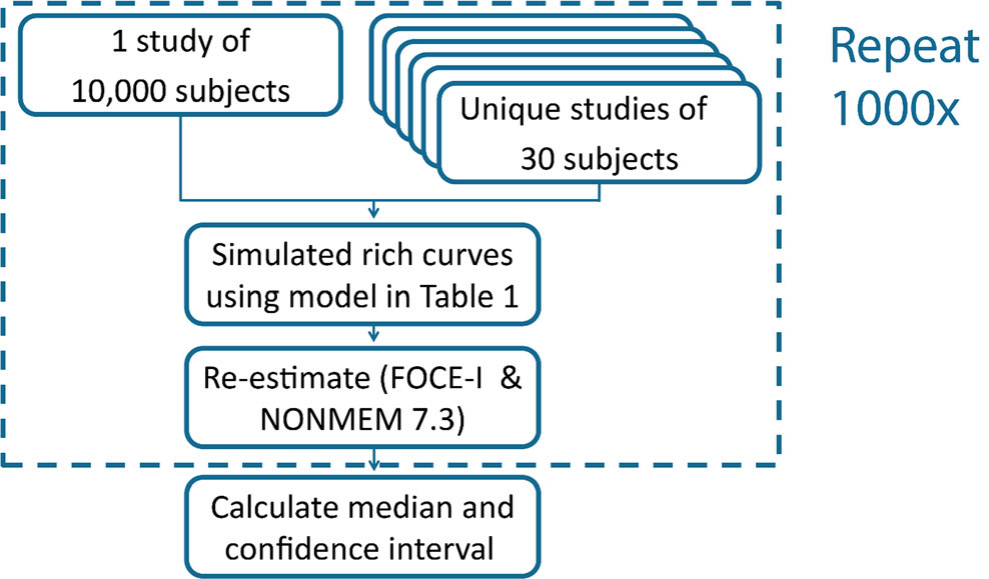
Flow chart of the analytical approach

After simulation, we re-estimated all parameters in each simulated dataset. The re-estimation was performed using the first-order conditional estimation method with interaction. For each study size, we simulated and re-estimated 1000 studies. For the typical study, we sampled new individuals from NHANES for each simulation, thereby making 1000 unique studies, to simulate differences in covariate distributions between studies. Figure 1 shows a flow chart of the analytical approach.

### Outcome

The estimates from each of the 1000 studies, for each virtual drug and study size were summarized using the median and the 2.5th and 97.5th percentile. In this work, we will refer to this estimation interval as the 95% confidence interval (95% CI). Summary statistics were calculated for the pharmacokinetic parameters (CL, *V*, IIV CL, IIV *V*, and the residual error) and for the estimates of F*fat*. The 95% CIs of the pharmacokinetic parameters were used to assess the reliability of the parameter estimates given the study design. To illustrate the change in precision with increasing sample size, we repeated the above method for study sizes of 60, 100, 150, 200, 250,500, 1000, and 5000 subjects with an F*fat* of 1 on CL and V.

## Results

### Reliability of pharmacokinetic parameter estimates

**Fig. 2:**
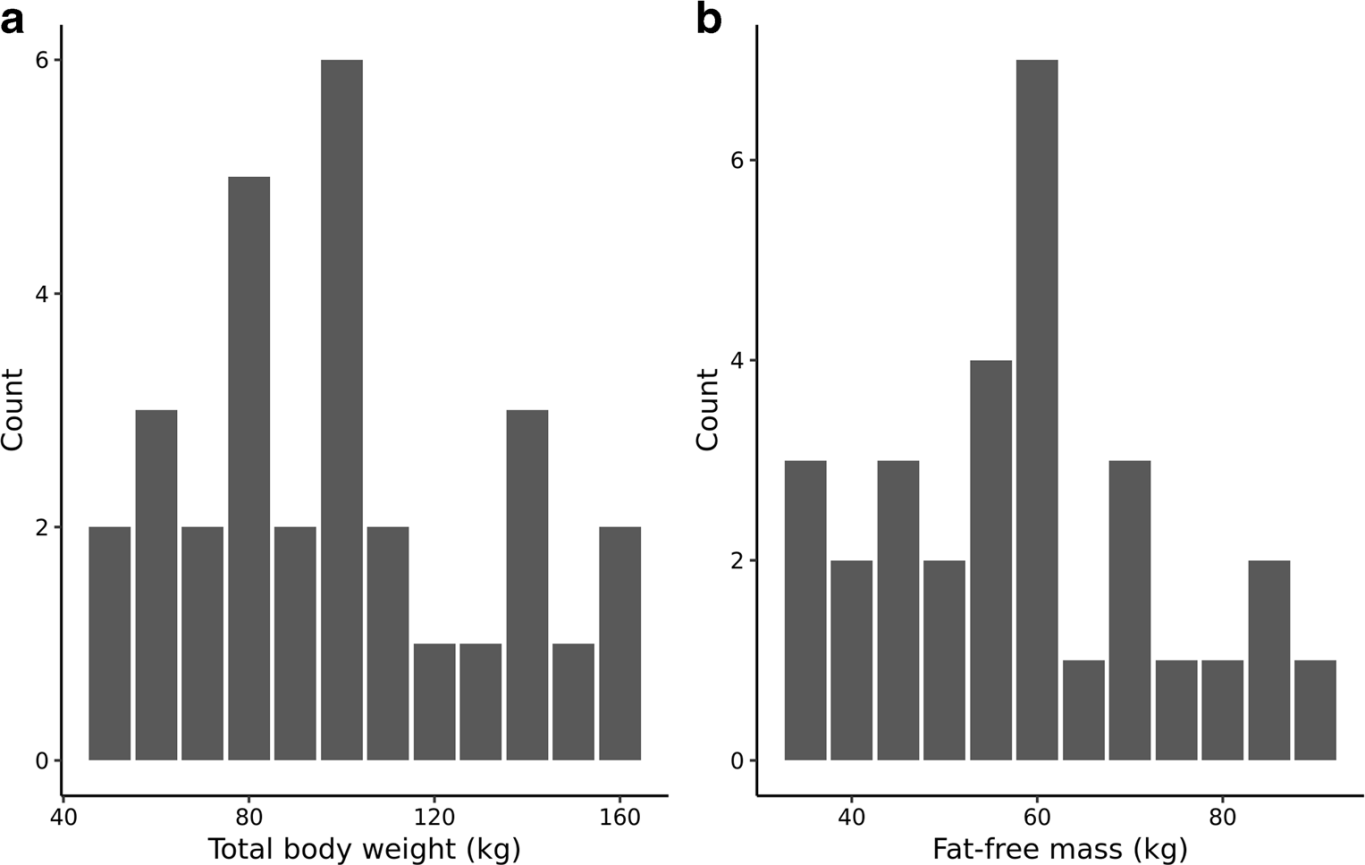
Distribution of total body weight (**a**) and fat-free mass (**b**) of a randomly sampled typical study containing 30 subjects, 10 subjects for each BMI group (non-obese, obese, and morbidly obese). For the figure, subjects were summarized in 10-kg weight bands for TBW and 5-kg weight bands for FFM

Figure 2 shows a histogram of TBW and FFM distribution of a randomly sampled study (out of 1000 studies), indicating a wide range of body size. Figure S1 in the supplemental materials shows the distribution of TBW and FFM in the large (*n* = 10,000) study. Table 1 shows the median and 95% CI for the typical study size of a virtual drug with F*fat* of 0.5 in NFM on both CL and V. All parameters were estimated with low bias (< 10%) and narrow 95% CI indicating that with our typical study size it is possible to accurately estimate the primary pharmacokinetic parameters. The precision and bias of the estimates of the primary pharmacokinetic parameters in the other 15 virtual drugs were similar (data not shown).

**Table 1:** Pharmacokinetic parameters of the one-compartment model and the estimates in the typical study size including a virtual drug with F*fa*t 0.5 in NFM on both CL and V

	True value	Median estimate	95% CI
Clearance (L/h)	0.69	0.71	0.63–0.78
Volume of distribution (L)	1.0	1.0	0.99–1.1
F*fat* on clearance	0.5	0.51	− 0.11–2.1
F*fat* on volume of distribution	0.5	0.48	− 0.06–1.6
Inter-individual variability			
Clearance (%)	30	28	20–36
Volume of distribution (%)	30	28	20–36
Residual error (%)	15	15	14–16

*95% CI*, 95% confidence interval. Clearance and volume of distribution were standardized on a male with a total body weight of 100 kg and a fat-free mass of 60 kg

### Reliability of F*fat* estimates in large studies

**Fig. 3:**
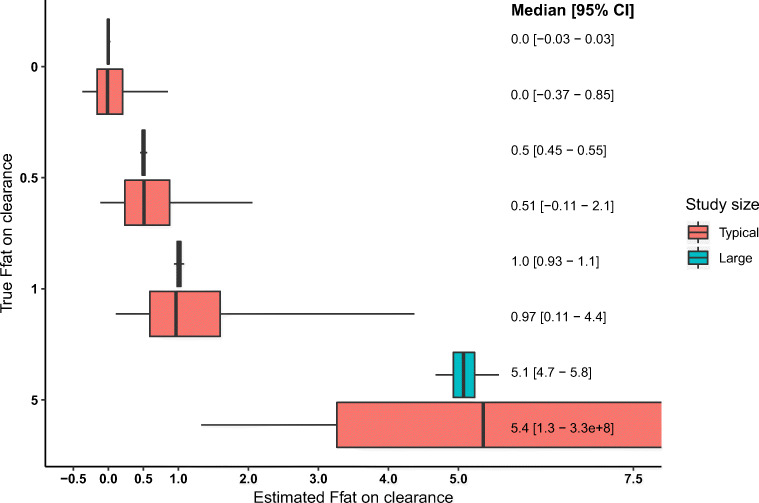
Median and 95% confidence intervals for F*fat* in NFM on clearance for a typical study size (*n* = 30; in red) and a large study size (*n* = 10,000; in cyan). The box represents the 25th and 75th percentile. The whiskers represent the 95% confidence interval. The gray shadow represents the true value of F*fat*

**Fig. 4:**
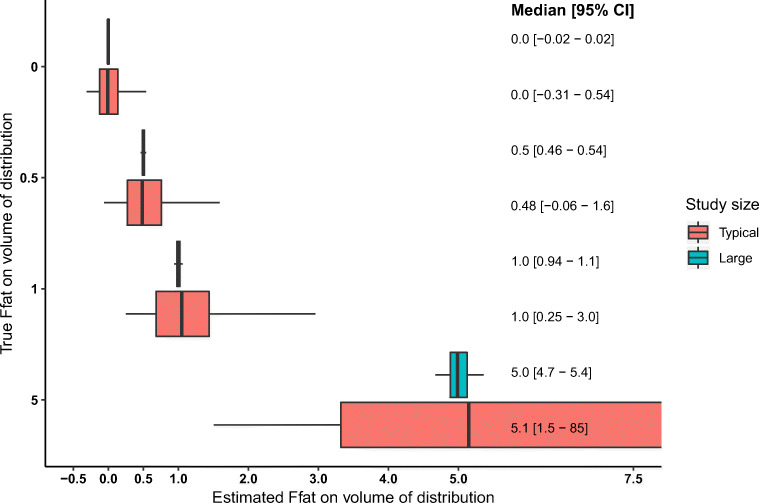
Median and 95% confidence intervals for estimated F*fat* on the volume of distribution for a typical study size (*n* = 30; in red) and a large study size (*n* = 10,000; in cyan). The box represents the 25th and 75th percentile. The whiskers represent the 95% confidence interval. The gray shadow represents the true value of F*fat*

We found a narrow 95% CI for F*fat* in all the 16 virtual drugs (Figs. 3 and 4 in cyan). This indicates that F*fat* can be estimated accurately and precisely when a large data set is available. The 95% CI of F*fat* in NFM on CL was independent of the F*fat* value in NFM on *V*, meaning that these could be estimated independently from each other regardless the value of the other estimate of F*fat* (data not shown). For example, the 95% CI the F*fat* on CL with a true value of 1 was 0.11–4.4 regardless of the (true) value of the F*fat* on *V* (i.e., whether F*fat* on V was 0, 0.5, 1, or 5). Therefore, we only show combinations for the large and the typical study size where F*fat* values were equal for CL and *V* (i.e. F*fat* of 1 in NFM on both CL and *V*).

### Reliability of F*fat* estimates in typical studies

We found a large 95% CI (Figs. 3 and 4 in red). The intervals of the different values of F*fat* were overlapping to a large extent which makes it challenging to interpret an estimated F*fat*. Interestingly, the lower range of the 95% CIs of the F*fat* values of 0 and 0.5 was below zero, which indicates for CL that an increasing amount of fat mass results in a lower CL which is opposite to the relation used to simulate the data. For the F*fat* value of 5 (representing the situation where fat mass is the most important metric driving individual variability in CL or V), the 95% CIs were very wide indicating that the fact that the estimate is a high value is more informative than the exact value itself. Finally, the 95% CIs of F*fat* 0 did not contain 1 and the 95% CI of 1 did not contain 0, indicating that for our study design, size and drug characteristics using NFM as an exploratory tool should result in a model closer to the true model compared to a priori choosing a fixed value of 0 (FFM) or 1 (TBW).

### Reliability of F*fat* estimates in other study sizes

**Fig. 5:**
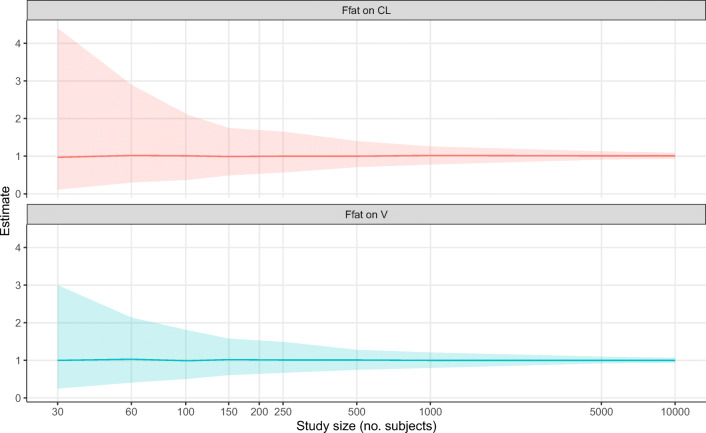
Median (line) and 95% confidence intervals (shade) for estimated F*fat* on clearance and volume of distribution with increasing study size where the true value of F*fat* on both clearance and volume of distribution is 1*.* Underlying data is presented in Table S1 in the supplemental materials

The precision of the estimates of F*fat* on CL and V of other study sizes is shown in Fig. 5. As can be expected, the more subjects that are included, the narrower the 95% CI. However, even with 100 subjects, the interval for the F*fat* estimate on CL and *V* is relatively large including both 0.5 and 2 in its interval. Increasing the study to 250 normal weight, obese and morbidly obese subjects only slightly improves the precision.

## Discussion

Although we showed that F*fat* is identifiable in (large) pharmacokinetic studies, we found that it cannot be reliably estimated in pharmacokinetic studies with a typical study size in an otherwise best-case scenario. In our simulation study, we found values of F*fat* in NFM on CL between − 0.1 and 2.1, while the true value was 0.5.

The median value of the estimates of 1000 replicated studies of any value of F*fat* is indeed accurate but for a single study, one never knows the true value. We show here that an incorrect value of F*fat* will be found regularly meaning that the estimate of F*fat* is study-specific if the power of the estimate is not evaluated. Using an inaccurate value of F*fat* could result in an erroneous prediction of the pharmacokinetics of a drug when extrapolated to another population.

In this simulation study, we mainly focused on two study sizes to illustrate the potential pitfall of F*fat*; hence, we did not study other study sizes or scenarios in detail. In Fig. 5, we show that increasing the sample size per study will decrease the confidence interval of F*fat* estimations. However, a general statement on the minimal study size to estimate F*fat* is not useful since study size is just one of the factors attributing to parameter identifiability and reliability. Other factors include the study design, number of observations, size of the inter-individual variability, size of the residual variability, the distribution of the covariates (e.g., uniform, normal or log-normal), and covariate effect size [22]. In our study, we chose a best-case scenario situation, of an intravenously administered drug, with rich sampling and low residual error. Therefore, in real-life situations, we expect that NFM cannot be reliably estimated. Furthermore, Fig. 5 illustrates that even in a best-case scenario setting and a very large sample size of 100 richly sampled individuals, there would only be a marginal improvement of the precision of the F*fat* estimates. Although our study was not designed to find the optimal design and sample size for estimation of F*fat*, considering the uniqueness of each drug and population, we nonetheless show that it is unlikely that F*fat* can be reliably estimated in a typical study. One may argue that a poorly designed study will always result in poor estimates. From our results, it can be argued that testing fixed estimates of F*fat* (0 for fat-free mass or 1 for total body weight) as size descriptors in a pharmacokinetic study may also result in selection of incorrect size predictors. Pharmacometricians should be aware of this phenomenon when performing pharmacokinetic studies. Future studies should investigate whether, for example, physicochemical properties of drugs may be used to predict the best size descriptor for allometric scaling in pharmacokinetic studies.

The NFM method is an elegant solution to investigate the contribution of fat mass to variability in pharmacokinetics. The NFM method can be very useful in a covariate analysis to investigate several size metrics in a single run after which the best one can be selected. The use of NFM has an advantage compared to traditional body size metrics, at the cost of only one parameter. However, its use is usually not preceded by careful study design and sample design evaluation, potentially leading to imprecise estimates. We have shown here that the point estimates of F*fat* have low precision in realistically sized pharmacokinetic studies, even in a best-case scenario. Consequently, the estimation and reporting of NFM in these studies should be performed with caution and, if used, should always be preceded by rigorous study design evaluation.

## Conclusion

The novel concept of NFM can be advantageous in sufficiently large studies but may have limitations in realistically sized pharmacokinetic studies, predominantly in reliability of the estimates F*fat*. This might have consequences for prediction of dosing of medications with a narrow therapeutic window in patients with extreme body sizes. We strongly advocate rigorous study design and sample size evaluation before applying this potentially very useful methodology in a pharmacokinetic study.

## Supplementary Information

ESM 1 (DOCX 81 kb)

## References

[1] McLeay SC, Morrish GA, Kirkpatrick CM, Green B (2012). The relationship between drug clearance and body size: systematic review and meta-analysis of the literature published from 2000 to 2007. Clin Pharmacokinet.

[2] Holford NHG, Anderson BJ (2017). Allometric size: the scientific theory and extension to normal fat mass. Eur J Pharm Sci.

[3] Cortinez LI, Anderson BJ, Penna A, Olivares L, Munoz HR, Holford NH (2010). Influence of obesity on propofol pharmacokinetics: derivation of a pharmacokinetic model. Br J Anaesth.

[4] Dorlo TP, Huitema AD, Beijnen JH, de Vries PJ (2012). Optimal dosing of miltefosine in children and adults with visceral leishmaniasis. Antimicrob Agents Chemother.

[5] Hopkins AM, Wojciechowski J, Abuhelwa AY, Mudge S, Upton RN, Foster DJ (2017). Population pharmacokinetic model of doxycycline plasma concentrations using pooled study data. Antimicrob Agents Chemother.

[6] Rhodin MM, Anderson BJ, Peters AM, Coulthard MG, Wilkins B, Cole M, Chatelut E, Grubb A, Veal GJ, Keir MJ, Holford NHG (2009). Human renal function maturation: a quantitative description using weight and postmenstrual age. Pediatr Nephrol.

[7] Salem AH, Giranda VL, Mostafa NM (2014). Population pharmacokinetic modeling of veliparib (ABT-888) in patients with non-hematologic malignancies. Clin Pharmacokinet.

[8] Tham LS, Wang LZ, Soo RA, Lee HS, Lee SC, Goh BC, Holford NHG (2008). Does saturable formation of gemcitabine triphosphate occur in patients?. Cancer Chemother Pharmacol.

[9] Wright DF, Stamp LK, Merriman TR, Barclay ML, Duffull SB, Holford NH (2013). The population pharmacokinetics of allopurinol and oxypurinol in patients with gout. Eur J Clin Pharmacol.

[10] Zvada SP, Denti P, Geldenhuys H, Meredith S, van As D, Hatherill M, Hanekom W, Wiesner L, Simonsson USH, Jindani A, Harrison T, McIlleron HM (2012). Moxifloxacin population pharmacokinetics in patients with pulmonary tuberculosis and the effect of intermittent high-dose rifapentine. Antimicrob Agents Chemother.

[11] Cortinez LI, Anderson BJ, Holford NH, Puga V, de la Fuente N, Auad H (2015). Dexmedetomidine pharmacokinetics in the obese. Eur J Clin Pharmacol.

[12] McCune JS, Bemer MJ, Barrett JS, Scott Baker K, Gamis AS, Holford NH (2014). Busulfan in infant to adult hematopoietic cell transplant recipients: a population pharmacokinetic model for initial and Bayesian dose personalization. Clin Cancer Res.

[13] Smythe W, Khandelwal A, Merle C, Rustomjee R, Gninafon M, Bocar Lo M, Sow OB, Olliaro PL, Lienhardt C, Horton J, Smith P, McIlleron H, Simonsson USH (2012). A semimechanistic pharmacokinetic-enzyme turnover model for rifampin autoinduction in adult tuberculosis patients. Antimicrob Agents Chemother.

[14] Allegaert K, Olkkola KT, Owens KH, Van de Velde M, de Maat MM, Anderson BJ (2014). Covariates of intravenous paracetamol pharmacokinetics in adults. BMC Anesthesiol.

[15] Anderson BJ, Holford NH (2008) Mechanism-based concepts of size and maturity in pharmacokinetics. Annu Rev Pharmacol Toxicol 48:303–332 10.1146/annurev.pharmtox.48.113006.094708 17914927

[16] Centers for Disease Control and Prevention (CDC), National Center for Health Statistics (NCHS). National Health and Nutrition Examination Survey Data. Hyattsville, MD: U.S. Department of Health and Human Services, Centers for Disease Control and Prevention, 2018. Available from: https://www.cdc.gov/nchs/nhanes. Accessed 14 Feb 2018

[17] Janmahasatian S, Duffull SB, Ash S, Ward LC, Byrne NM, Green B (2005). Quantification of lean bodyweight. Clin Pharmacokinet.

[18] Lindbom L, Pihlgren P, Jonsson EN (2005). PsN-Toolkit--a collection of computer intensive statistical methods for non-linear mixed effect modeling using NONMEM. Comput Methods Prog Biomed.

[19] Beal S, Sheiner LB, Boeckmann A, RJ B. NONMEM User’s Guides. (1989–2009). Ellicott City, MD, USA. 2009

[20] European Medicine Agency. Guideline on bioanalytical method validation. July, 2011 10.4155/bio.12.44 22533559

[21] Food and Drug Administration. Bioanalytical method validation - guidance for industry. May, 2018

[22] Ribbing J, Jonsson EN (2004). Power, selection bias and predictive performance of the population pharmacokinetic covariate model. J Pharmacokinet Pharmacodyn.

