# Supplementary material for: Normal fat mass cannot be reliably estimated in typical pharmacokinetic studies
Source: Eur J Clin Pharmacol. 2020 Nov 18;77(5):727–33. doi: 10.1007/s00228-020-03042-4 (PMC8032617; doi:10.1007/s00228-020-03042-4)
Supplement: Supplementary file 1 — (DOCX 81 kb) [file 228_2020_3042_MOESM1_ESM.docx]

Normal fat mass cannot be reliably estimated in typical pharmacokinetic studies

Roeland E. Wasmann^a^, Elin M. Svensson^a,b^, Stein J. Schalkwijk^a^, Roger J. Brüggemann^a^, Rob ter Heine^a^

^a^ Department of Pharmacy, Radboud Institute for Health Sciences, Radboud University Medical Center, Nijmegen, The Netherlands,
^b^ Department of Pharmaceutical Biosciences, Uppsala University, Uppsala, Sweden

# SUPPLEMENTS

**Simulation model code**

$PROB NFM SIMULATIONS

$INPUT C ID TIME AMT DV MDV EVID CMT WT FFM

$DATA DATAFILE.CSV IGNORE=@

$SUBROUTINE ADVAN1 TRANS1

$MODEL COMP (CENTRAL)

$PK

MED_FFM = 60 ; Median fat-free mass

MED_WT = 100 ; Median total body weight

; Calculation of median NFM used for standardization

MED_NFM_CL = MED_FFM + THETA(3)*(MED_WT-MED_FFM)

MED_NFM_V = MED_FFM + THETA(4)*(MED_WT-MED_FFM)

; Individual NFM for clearance and volume of distribution

NFM_CL = FFM+THETA(3)*(WT-FFM)

NFM_V = FFM+THETA(4)*(WT-FFM)

; Allometric scaling of clearance and volume of distribution

ALLO_CL = (NFM_CL/ MED_NFM_CL)**0.75

ALLO_V = (NFM_V/ MED_NFM_V)

; Typical and individual parameters

TVCL = THETA(1) ; Typical value for clearance

TVV1 = THETA(2) ; Typical value for volume of distribution

CL=TVCL*ALLO_CL*EXP(ETA(1)) ; Individual clearance

V1=TVV1*ALLO_V*EXP(ETA(2)) ; Individual volume of distribution

S1=V1

K10=CL/V1 ;

$ERROR

IPRED = F

Y=IPRED*(1+ERR(1))

$THETA

0.693 ; Clearance (L/h)

1 ; Volume of distribution (L)

0.5 ; Ffat for clearance – other values for other simulations

0.5 ; Ffat for volume of distribution – other values for other simulations

$OMEGA

0.08618 ; Inter individual variability on clearance

0.08618 ; Inter individual variability on volume of distribution

$SIGMA

0.02225 ; Proportional error

$ESTIMATION METH=1 INTERACTION MAXEVAL=2000 PRINT=5 NSIG=3 SIGL=9

Table S1. Median and 95% Confidence interval for F*fat* on CL and F*fat* on V where the true value was 1 for different study size.

| **Study size**  **n** | **F*fat* on CL**  Median [95% CI] | **F*fat* on CL**  Median [95% CI] |
| --- | --- | --- |
| 30 | 0.97 [0.11 – 4.4] | 1.00 [0.25 – 3.0] |
| 60 | 1.02 [0.30 – 2.90] | 1.03 [0.41 – 2.14] |
| 100 | 1.01 [0.37 – 2.10] | 0.99 [0.51 – 1.80] |
| 150 | 0.99 [0.49 – 1.75] | 1.02 [0.61 – 1.58] |
| 200 | 1.01 [0.55 – 1.74] | 1.02 [0.64 – 1.56] |
| 250 | 1.00 [0.57 – 1.65] | 1.01 [0.67 – 1.49] |
| 500 | 1.00 [0.71 – 1.40] | 1.01 [0.75 – 1.28] |
| 1,000 | 1.02 [0.78 – 1.26] | 1.00 [0.80 – 1.21] |
| 5,000 | 1.01 [0.91 – 1.13] | 1.00 [0.92 – 1.10] |
| 10,000 | 1.01 [0.93 – 1.09] | 1.00 [0.94 – 1.07] |


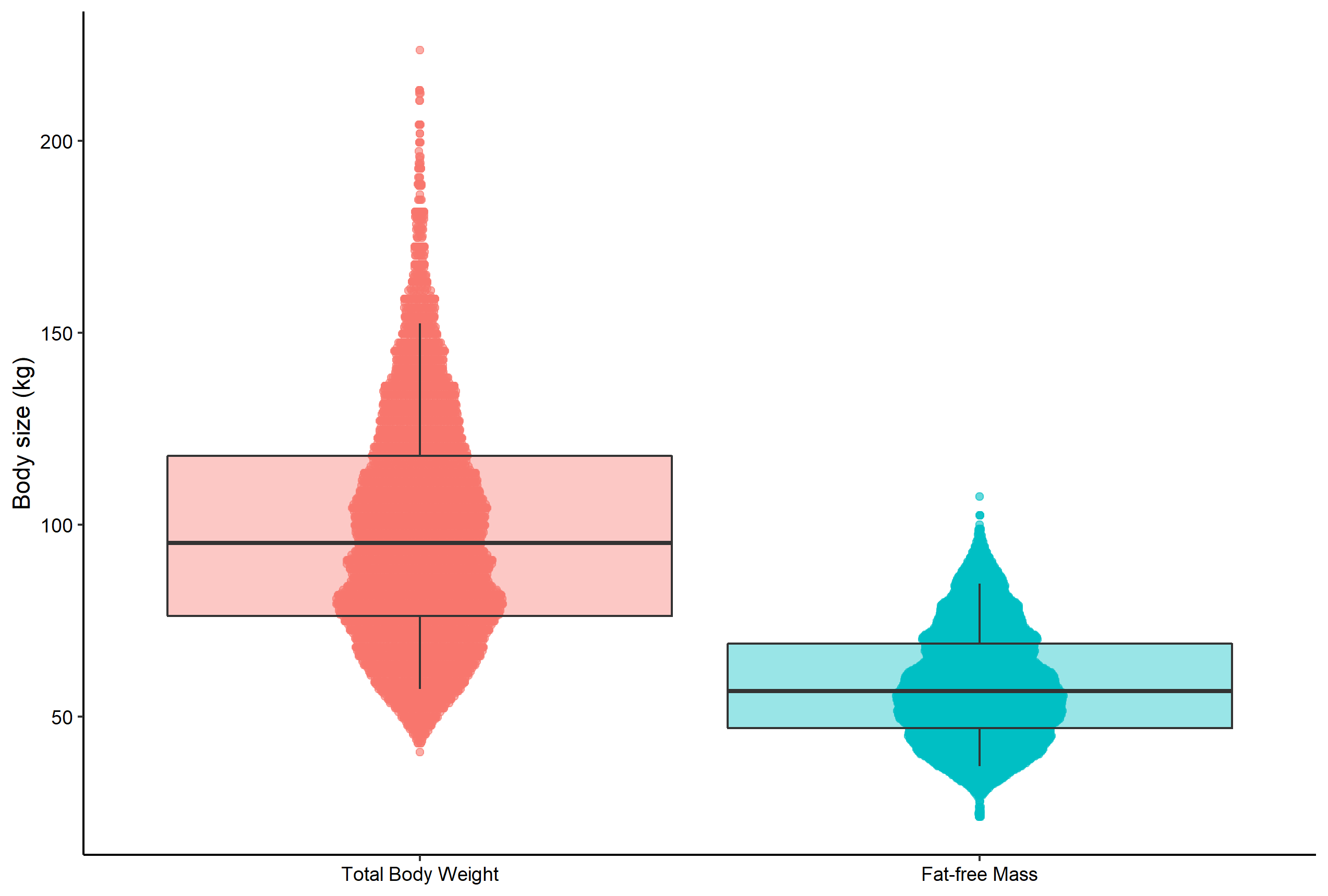


Figure S1. Distribution of total body weight (A) and fat-free mass (B) of the large study containing 10,000 subjects. The box represents the 25th and 75th percentile, the whiskers represent the 2.5^th^ and 97.5^th^ percentiles. The individual points are arranged to show the probability density.
